# Tissue-Specific Upregulation of MDS/EVI Gene Transcripts in the Intestine by Thyroid Hormone during *Xenopus* Metamorphosis

**DOI:** 10.1371/journal.pone.0055585

**Published:** 2013-01-31

**Authors:** Thomas C. Miller, Guihong Sun, Takashi Hasebe, Liezhen Fu, Rachel A. Heimeier, Biswajit Das, Atsuko Ishizuya-Oka, Yun-Bo Shi

**Affiliations:** 1 Section on Molecular Morphogenesis, Program in Cellular Regulation and Metabolism (PCRM), Eunice Kennedy Shriver National Institute of Child Health and Human Development (NICHD), National Institutes of Health (NIH), Bethesda, Maryland, United States of America; 2 Key Laboratory of Allergy and Immune-related Diseases and Centre for Medical Research, School of Medicine, Wuhan University, Wuhan, People's Republic of China; 3 Department of Biology, Nippon Medical School, Kosugi-cho, Kawasaki, Kanagawa, Japan; University Claude Bernard Lyon 1, France

## Abstract

**Background:**

Intestinal remodeling during amphibian metamorphosis resembles the maturation of the adult intestine during mammalian postembryonic development when the adult epithelial self-renewing system is established under the influence of high concentrations of plasma thyroid hormone (T3). This process involves de novo formation and subsequent proliferation and differentiation of the adult stem cells.

**Methodology/Principal Findings:**

The T3-dependence of the formation of adult intestinal stem cell during *Xenopus laevis* metamorphosis offers a unique opportunity to identify genes likely important for adult organ-specific stem cell development. We have cloned and characterized the ectopic viral integration site 1 (EVI) and its variant myelodysplastic syndrome 1 (MDS)/EVI generated via transcription from the upstream MDS promoter and alternative splicing. EVI and MDS/EVI have been implicated in a number of cancers including breast, leukemia, ovarian, and intestinal cancers. We show that EVI and MDS/EVI transcripts are upregulated by T3 in the epithelium but not the rest of the intestine in *Xenopus laevis* when adult stem cells are forming in the epithelium.

**Conclusions/Significance:**

Our results suggest that EVI and MDS/EVI are likely involved in the development and/or proliferation of newly forming adult intestinal epithelial cells.

## Introduction

Amphibian metamorphosis resembles the postembryonic development around birth in mammals with both processes taking place when plasma thyroid hormone (T3) concentrations are high [1]–[3]. Furthermore, both metamorphosis and mammalian postembryonic development involve the formation/maturation of adult organs and thus the establishment of the adult stem cell systems critical for tissue renewal and replacement in the adult. These similarities and the ability to manipulate amphibian metamorphosis by simply controlling the availability of T3 to the tadpoles make amphibian metamorphosis an excellent model to investigate the formation of adult organ-specific stem cells [4], [5], an area poorly studied. During *Xenopus laevis* (*X. laevis*) metamorphosis, remodeling of the intestine occurs via extensive apoptosis of larval epithelial cells followed by *de novo* formation and subsequent proliferation and differentiation of adult stem cells to form the adult intestine [6], [7]. Just like other processes during metamorphosis, this remodeling of the intestine is wholly dependent upon T3 and the formation of the mature frog intestine appears to be largely analogous, both morphologically and genetically, to the postembryonic maturation of the mammalian intestine when plasma T3 is likewise elevated [4], [5], [8]–[10]. However, the dependence of the mammalian fetus and neonates on maternal nutrients, trophic factors, and hormones can make it difficult to tease apart the endogenous pathways induced in the offspring from ones initiated or maintained by the mother. Thus, *X. laevis* is a unique and powerful model to determine how T3 controls the developmental formation of adult intestinal stem cells, which in turn could lead to better identification of deregulated pathways that lead to cancer and other diseases.

Abnormal expression of the transcription factor ectopic viral integration site 1 (EVI) and its variant myelodysplastic syndrome 1 (MDS)/EVI, generated from the alternative splicing of the transcript of the MDS gene that linked the exon 2 of MDS to the exon 2 of EVI, have been implicated in a number of epithelial cancers and directly linked to severity of breast, leukemia, ovarian, and intestinal cancers [11]–[16]. On the other hand, the developmental and physiological function of EVI and MDS/EVI, especially in organ-specific stem cells, has been largely unexplored outside of its role in hematopoietic stem maintenance [17]–[19]. We have recently identified an EST encoding a region of the 3′-UTR of the EVI genes from a cDNA microarray analysis as a transcript that is strongly regulated in the epithelium of the intestine during metamorphosis (unpublished observation). To further investigate the possible involvement of EVI in intestinal development, we have cloned the *Xenopus* MDS/EVI and MDS transcripts. We found that like in human, both EVI and MDS/EVI transcripts exist. Making use of the existence of the sequenced genome of *X. tropicalis*, a highly related *Xenopus* species, we showed that human and *Xenopus* MDS/EVI locus is highly conserved, although we failed to clone or identify the homologous region of the last exon (exon 3) of human MDS in either *Xenopus* species. More importantly, our expression studies showed that all three transcripts: MDS, EVI, and MDS/EVI, were produced in the intestine of *X. laevis* and that they were coregulated with their expression peaked at the metamorphic climax in the intestinal epithelium, coinciding with adult stem cell development. Moreover, the expression of these genes was strongly induced by T3 treatment of premetamorphic tadpoles. Taken together, this suggests EVI and MDS/EVI may play an important role in the induction, proliferation, and/or differentiation of adult stem cells in the intestine.

## Materials and Methods

### Experimental animals

All experiments involving *X. laevis* animals were carried out as approved by the Animal Use and Care Committee of Eunice Kennedy Shriver National Institute of Child Health and Human Development, National Institutes of Health. *X. laevis* adults were purchased from NASCO (Fort Atkinson, WI). Tadpoles of *X. laevis* were purchased from NASCO or produced and reared in the laboratory. The developmental stages of tadpoles were assigned as published [20]. When indicated, premetamorphic tadpoles at stage 54 were treated with 5 nM T3 for 0 –7 days.

### Cloning MDS, and MDS/EVI cDNA

**Figure 1:**
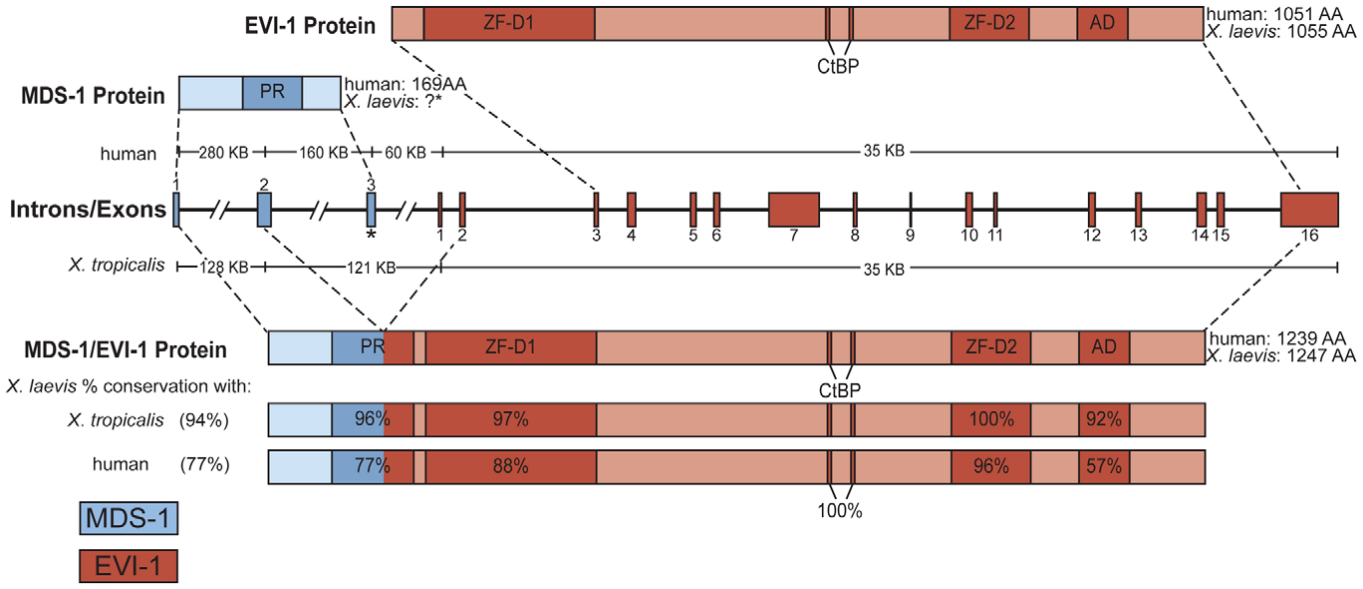
Structure and conservation of EVI and MDS in humans, *X. laevis*, and *X. tropicalis*. There are 16 exons for human and *X. tropicalis* EVI and 3 exon for human MDS. In *X. laevis*, as in humans, exon 2 of MDS can be spliced to exon 2 of EVI to form a larger, fusion transcript MDS/EVI. The % identity of the PR (PRDI-BF1 and RIZ homology domain containing) domain, zinc finger domains (ZF-D) 1 and 2, C-terminal binding protein (CtBP) domain, and acidic domain (AD) are indicated. For an alignment of the EVI amino acid sequence, see [21]. Note that despite an exhaustive search by using RACE and *in silico* methods, no MDS exon 3 could be found in *X. laevis* or *X. tropicalis*, although a 3′ end of MDS exon 2 that is not present in MDS/EVI fusion transcript was cloned by using RACE (not shown). Thus, the existence of the MDS transcript and encoding protein remains unknown as indicated with a question mark in the figure.

*X. laevis* stage 62 whole intestinal RNA was purified by using TRIZOL (Invitrogen, Grand Island, NY) or SV Total RNA Isolation System (Promega, Madison, WI) treated with RNase free DNase I (Invitrogen) and then cDNA generated using the High Capacity Reverse Transcription Kit (Applied Biosystems, Foster City, CA). The full length human MDS sequence was BLAST against *X. tropicalis*, a species highly related to *X. laevis*, and a forward primer (5′-TCACCCTACAAAGCACCAATTTAC-3′) within a conserved region of the predicted *X. tropicalis* MDS exon 2 was synthesized and paired with a reverse primer (5′-CAGCAAATGCTTCTCCAGGCTTTGC-3′) that overlapped *X. laevis* EVI exon 5 and 6 (Fig. 1) to produce a PCR product (Ex Taq polymerase, Takara Bio Inc., Shiga, Japan). That product was cloned and transformed into competent bacteria using TOPO TA Cloning Kit (Invitrogen), grown in culture, plasmid purified using a spin Mini-Prep kit (Qiagen, Valencia, CA), and sequenced (MWG Operon, Huntsville, AL). Using a reverse (5′-TGGCCCAAACTTCTCTCCAACCGA-3′) primer designed from the thus identified *X. laevis* MDS exon 2 sequence, 5′ MDS-specific rapid amplification of cDNA ends (RACE)-ready cDNA was generated (Gene Racer RACE Ready cDNA Kit, Invitrogen). Then using forward primers provided by the kit and reverse primers specific to *X. laevis* MDS exon 2 (5′-GATTCCAAGTCCTGTGCCTGGGATG-3′, 5′-CTGATGGAATAGGGATGTCGTCTGG-3′) nested PCRs were used to obtain a clone that was subsequently sequenced to determine the full-length sequence for MDS/EVI. Three different primers (5′-AGCTGCACCGGGGAACTGACACCA-3′, 5′-ATGAGATGCAAAGGCAGGGCAAGG-3′, 5′-TCACCCTACAAAGCACCAATTTAC-3′) were used for 3′ RACE in an attempt to identify full length MDS mRNA. As a result, we obtained an additional sequence after the 3′ end of MDS exon 2 but failed to clone any exon that corresponds to the MDS exon 3 of human MDS. The sequences have been submitted to GenBank with the accession numbers JX871943 for *X. laevis* MDS/EVI and JX871943 for *X. laevis* MDS. Functional domains were determined previously for *X. laevis* EVI [21] and the PR domain was determined from the homologous region of human MDS/EVI (also known as PRDM3) [22].

### Stage- and tissue-specific expression of EVI, MDS, and MDS/EVI

The anterior region of the small intestine from tadpoles at prometamorphic stage 56, metamorphic climax stage 61, and end of metamorphosis (stage 66), was dissected, flushed of contents, and treated with 1000 U/ml dispase (Godo, Tokyo, Japan) at 37°C for 30 min to separate the epithelium (Ep) from non-epithelial tissues (non-Ep), which is mostly connective tissue and muscles [23]. Total RNA was isolated from these tissues or total intestine at indicated stages with or without T3 treatment.

Quantitative real-time PCR (qPCR) was carried out using SYBR Green PCR Mix and either Step One Plus Real-Time PCR System or ABI 7000 (Applied Biosystems, Foster City, CA) to determine the expression of transcripts relative to elongation factor 1 α (EF1α) expression as previously described [24], [25]. One reverse primer common for MDS/EVI and EVI was generated from EVI exon 2 (5′-TGGCAGGCGACCAGATTGGCTTGA-3′) while the MDS/EVI (5′-TCCCCAGTTTGGGTGGGAGGTCCTGG-3′, MDS exon 2) and EVI (5′-CAACCGGAACCCCTGTCGGACTGAG-3′, EVI exon 1) forward primers were transcript specific. Unique forward (5′-GGGCCATTTGAAGGAGAGCAATGCAT-3′) and reverse (5′-AACTGCATGAGGCACTGCACACTTTC-3′) primers were designed for quantitating MDS, with the reverse primer located in the novel 3′ portion of MDS exon 2 not present in the mature MDS/EVI mRNA.

When indicated, total expression of EVI and MDS/EVI was analyzed by using the primer set 5′- AGCACCATACCTGCCAGCATTC -3′ (forward) and 5′- ATAACCTGCCAACTGTCCCCAG -3′ (reverse), which is common for both EVI and MSD/EVI. Again, the expression level was normalized against EF1α expression.

T-tests (two populations) were performed to show levels of expression significantly lower than the peak in gene expression within each experiment as determined by triplicate qPCR analysis of samples. All qPCR experiments were repeated with individual sets of RNA to ensure reproducibility (data not shown).

## Results

### *Xenopus* and human MDS/EVI are highly conserved

In an effort to identify genes that are involved in the formation of adult intestinal stem cells during metamorphosis, we carried out a microarray analysis of genes regulated in the epithelium (Ep) or the rest (non-Ep) of the intestine during *X. laevis* metamorphosis. While the data is still being analyzed, we observed that a fragment within the 3′-UTR of the *Xenopus* homolog of human EVI gene was strongly upregulated at the climax of metamorphosis specifically in Ep but not non-Ep. The human EVI is known to be associated with a number of epithelial cancers including intestinal cancers and can also form a fusion protein MDS/EVI through alternative splicing of the MDS transcript onto the second exon of EVI. To investigate the existence of EVI and MDS/EVI in *Xenopus*, we made use of the existence of the published *Xenopus laevis* EVI sequence [21] and the availability of EVI genomic locus in *X. tropicalis*, a highly related *Xenopus* species, to carry out a series of 5′- and 3′- RACE analyses to clone the *X. laevis* MDS and MDS/EVI fusion transcripts. We successfully obtained the MDS/EVI cDNA (Fig. 1). Despite our repetitive effort, we failed to identify the equivalent of the 3^rd^ exon of human MDS gene, although we did obtained a version of the MDS transcript that extended past the end of exon 2 and was homologous to the intron region immediately after exon 2 of the MDS gene in *X. tropicalis* (not shown), suggesting that it likely represents a splicing intermediate. By using bioinformatics analysis on the *X. tropicalis* genomic sequence, we obtained the putative EVI and MDS/EVI regions with highly conserved organizations as the human genes (Fig. 1). Interestingly, no equivalent of the 3^rd^ exon of human MDS was identified in the *X. tropicalis* genome either, suggesting that either the 3^rd^ exon of MDS in *X. laevis or X. tropicalis* is absent or very different from the human gene. Thus the existence of the transcript encoding a homolog of human MDS, with the stop codon present in the 3^rd^ exon, remains to be determined.

The predicted EVI and MDS/EVI protein organizations in *X. laevis or X. tropicalis* are highly conserved to the human homologs. All EVI proteins in the three species contain two zinc finger domains that share over 88% identity (Fig. 1). They also contain an acidic domain that is over 92% identical between *X. laevis* and *X. tropicalis* and 57% between human and *X. laevis*. The MDS/EVI fusion protein contains these conserved domains plus an additional domain, the PRDI-BF1 and RIZ homology domain containing (PR) domain, which is also very conserved among the three species (Fig. 1). Thus, human and *Xenopus* EVI and MDS/EVI have conserved primary sequences and organizations, suggesting conserved functions.

### MDS, MDS/EVI, and EVI transcripts are regulated similarly during intestinal metamorphosis *in X. laevis*

**Figure 2:**
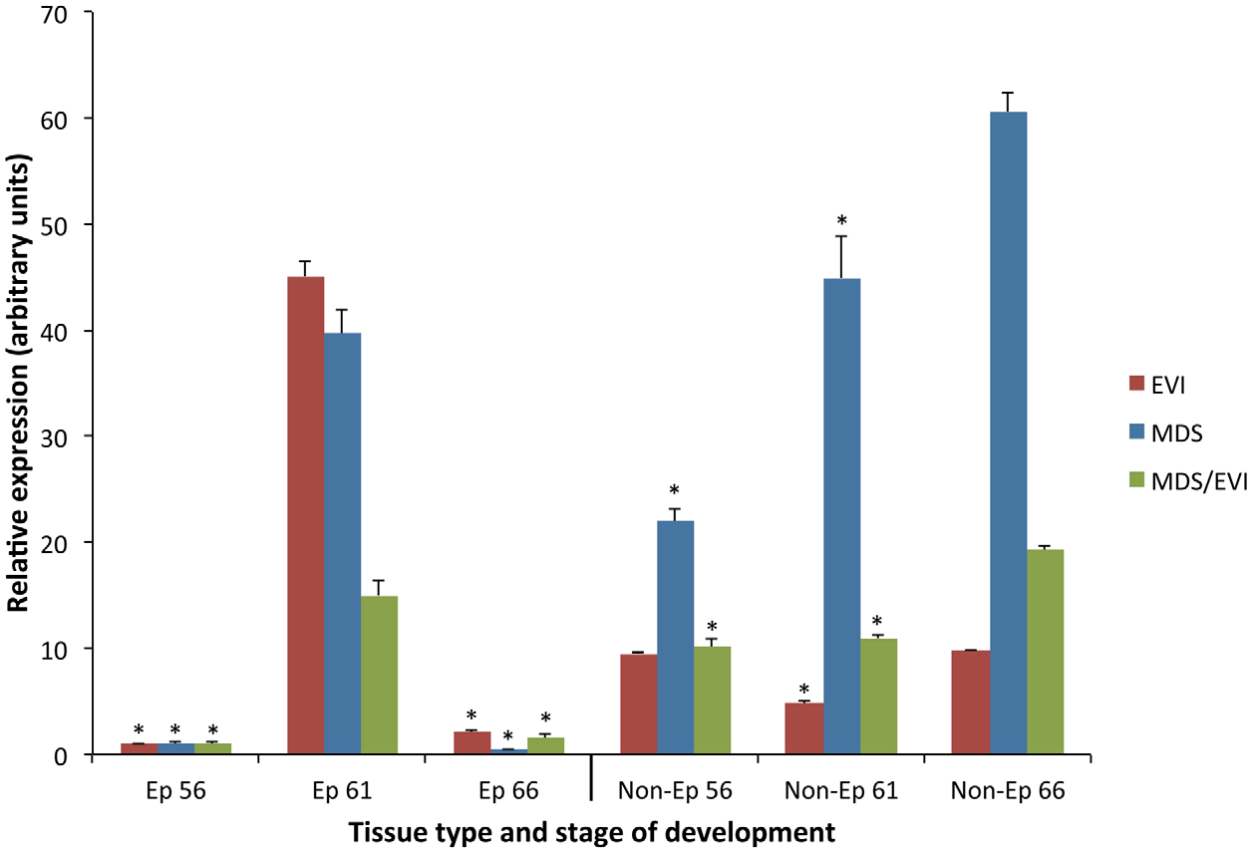
Tissue specific upregulation of EVI, MDS, and MDS/EVI transcripts in the intestine during metamorphosis. The expression of the transcripts was analyzed by using transcript-specific primer sets with qPCR on total RNA from the isolated intestinal epithelium (Ep) or the rest of the intestinal tissues (Non-Ep) of tadpoles at prometamorphosis (stage 56), the climax of metamorphosis (stage 61), and the end of metamorphosis (stage 66). The relative expression was obtained after normalizing against the control gene EF1α and was presented with the level at Ep 56 set to 1, allowing for easy comparison within each transcript. However, these data do not allow for comparisons of the expression levels between different transcript variants. Error bars represent the SEM (n = 3). * indicates transcript levels lower than the peak expression of the specific transcript in the indicated tissue (p≤0.05).

To investigate the potential involvement of EVI and MDS/EVI during metamorphosis, we designed primer sets unique to the three likely transcripts from the MDS and EVI genes. We isolated total RNA from either the Ep or non-Ep of the intestine at three different stages during metamorphosis. Stage 56 is a prometamorphic stage when endogenous T3 first becomes detectable (thus sometimes, it is also referred to as the onset of metamorphosis) and when the intestine is the typical premetamorphic intestine made of mostly larval intestinal epithelial cells surrounded by sparse connective tissue and muscles [6]. Stage 61 is the climax of metamorphosis when most larval epithelial cells undergo apoptosis and adult epithelial stem cells are formed de novo and rapidly proliferating, with concomitant development of the connective tissue and muscles. Stage 66 is the end of metamorphosis when a multiply folded adult epithelium is formed and surrounded by elaborate connective tissue and muscles. As shown in Fig. 2, all three transcripts had little expression in the Ep before or after metamorphosis but were strongly upregulated at the climax of metamorphosis (stage 61) when adult stem cells are formed and rapidly proliferating. In the non-Ep, all were expressed through out metamorphosis at fairly constant levels during metamorphosis, except MDS which showed a gradual increase (Fig. 2). Interestingly, at the climax of metamorphosis (stage 61), the ratio of the expression level of EVI in the Ep to that in the non-Ep was higher than those for MDS or MDS/EVI (Fig. 2), suggesting distinct regulation of the EVI and MDS promoter in the Ep and non-Ep.

### T3 upregulates the expression of EVI, MDS, and MDS/EVI in the intestine

**Figure 3:**
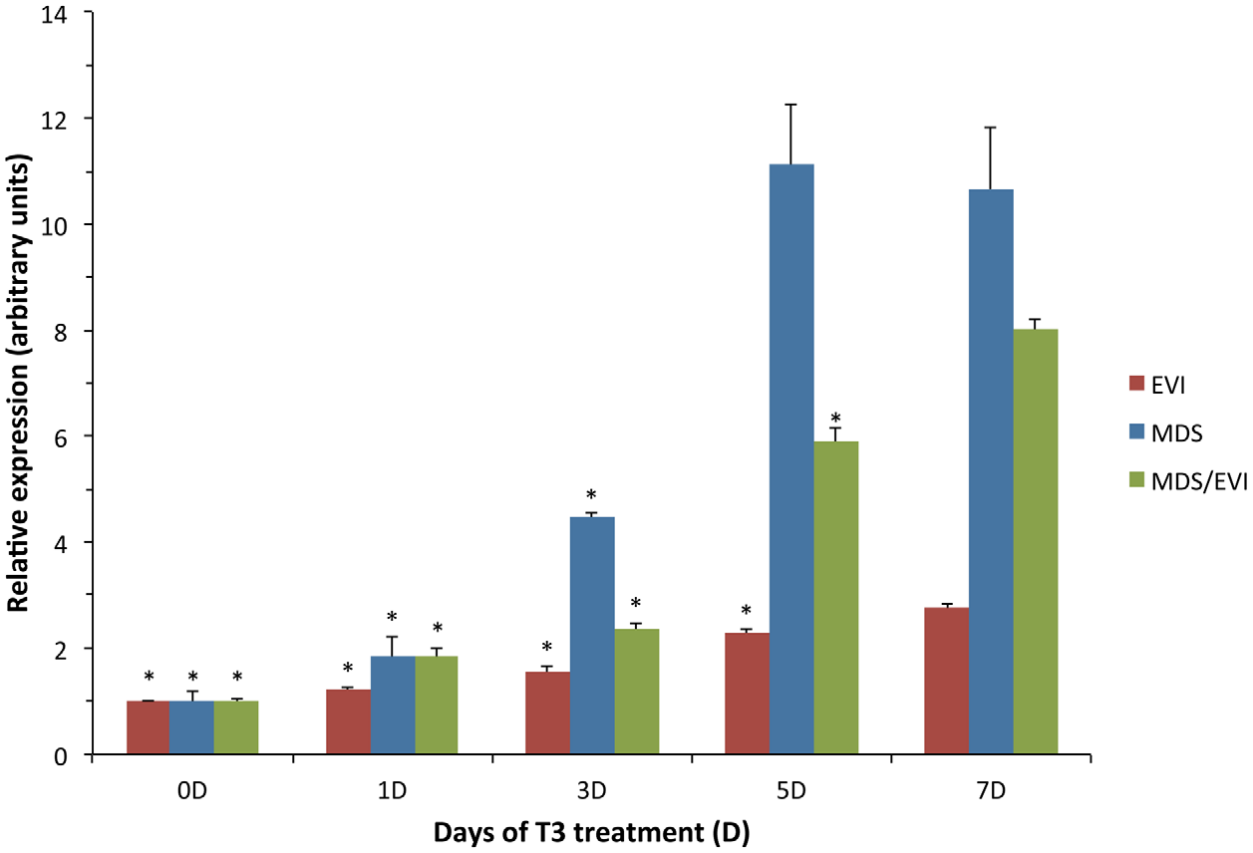
EVI, MDS, and MDS/EVI transcripts are induced in the intestine of premetamorphic tadpoles treated with T3. The expression of the transcripts was analyzed by using transcript-specific primer sets (as in Fig. 2.) on total intestinal RNA from tadpoles at stage 54 exposed to 5 nM T3 for 0–7 days. Error bars indicate SEM (n = 3). * indicates transcript levels lower than the peak expression of the specific transcript (p≤0.05).

As T3 controls the changes in all organs during metamorphosis, the correlation of EVI, MDS, and MDS/EVI expression with intestinal stem cell development suggests that their expression is regulated by T3. To test this possibility, we treated premetamorphic tadpoles at stage 54 with 5 nM T3, about the peak plasma level during natural metamorphosis [26], and isolated total RNA from the intestine for qRT-PCR analysis of EVI and MDS/EVI expression with primer sets recognizing individual transcripts. As shown in Fig. 3, their expression showed gradual increases in expression after 1 day of T3 treatment and peaked by 7 days. It should be pointed out that at the end of such a treatment, the intestinal epithelium resembles that at the climax of metamorphosis when most of the cells are proliferating adult progenitor/stem cells [27]. Expectedly, the down-regulation of EVI and EVI/MDS in the intestine at the end of metamorphosis was not observed during the T3-treatment due to the relatively short treatment period. Thus, T3 regulates the expression of EVI, MDS, and EVI/MDS during metamorphosis but it remains unclear if these transcript variants are direct or indirect response genes.

### EVI and MDS/EVI expression peaks at the climax of intestinal remodeling

**Figure 4:**
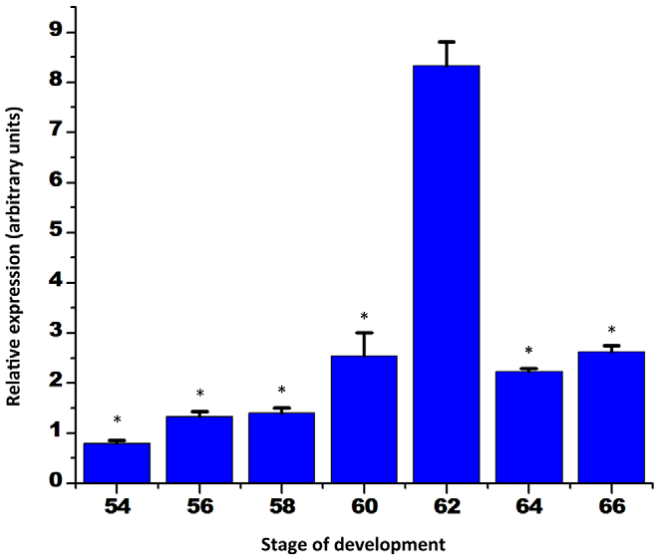
MDS/EVI is strongly upregulated in the intestine at the climax of metamorphosis. A primer set common to EVI and MDS/EVI was used for qPCR analysis on total intestinal RNA at different stages during metamorphosis as in Fig. 2. Note that the data is consistent with that in Fig. 2 if one considers the fact that there is little non-Ep in premetamorphic tadpoles at stage 56 but non-Ep increases as a percentage of the total intestine during metamorphosis. Error bars indicate SEM (n = 3). * indicates transcript levels lower than the peak expression (p≤0.05).

To investigate the developmental expression in the whole intestine, we then carried out qRT-PCR analysis on total intestinal RNA from different stages from premetamorphic to the end of metamorphosis. Given the similar temporal regulation pattern of the three transcripts within each tissue type, the expression levels of EVI and MDS/EVI in the total intestine in this and subsequent experiments were determined by using a primer set recognizing both EVI and MDS/EVI. As shown in Fig. 4, the expression was low in premetamorphic stages 54–56 and early metamorphic climax (stage 58) when plasma T3 is half of the maximal level observed during metamorphosis [26]. It increased dramatically by stage 60, when larval cell death occurs and adult epithelial stem cells become detectable [6]. The expression reached a maximal level at stage 62 (Fig. 4), when most of the larval epithelial cells had undergone apoptosis and were replaced by proliferating adult epithelial progenitor/stem cells [28]. Subsequently, as the adult epithelial progenitor/stem cells differentiated to form the adult epithelium during stages 64–66 [29], the expression was reduced. These temporal expression profiles suggest that the expression of EVI and MDS/EVI correlates with adult epithelial progenitor/stem cell formation and/or proliferation.

## Discussion

The adult vertebrate intestine has long been used as a model to study the property and function of adult stem cells in tissue repair and regeneration due to the constant self-renewal throughout adult life [6], [30]–[33]. The establishment of the self-renewing system of the intestinal epithelium takes place during the postembryonic developmental period when T3 levels are high. However, the underlying mechanisms regulated by T3 to induce these changes have yet to be determined. The remodeling of the intestine during amphibian metamorphosis mimics the formation of the adult epithelial renewing system in mammals both morphologically and molecularly, with many of the same genes upregulated during this period [4], [7], [10], [34]–[36]. Making use of the total T3-dependence of amphibian metamorphosis, we have identified here EVI and MDS/EVI as candidates involved in the formation and/or proliferation of the adult intestinal stem cells.

The human EVI is encoded by the EVI gene while MDS/EVI is generated via transcription from the MDS gene that is alternatively spliced to link exon 2 of the MDS gene to exon 2 of the EVI gene. In addition, the human MDS gene also produces an mRNA with three exons without any of the EVI exons. Both EVI and MDS/EVI transcripts are also present in *X. laevis* and *X. tropicalis*. On the other hand, we failed to either clone the equivalent of human MDS exon 3 in *X. laevis* or identify it in the genome of *X. tropicalis*, even though the genomic organization of the MDS/EVI locus is highly conserved between human and *X. tropicalis*. Thus, it is likely that either the MDS exon 3 is not conserved or absent in *Xenopus*. The high degree of conservation in both the genomic organization and predicted amino acid sequences of EVI and MDS/EVI between *Xenopus* and human argue that EVI and MDS/EVI have conserved functions.

The tissue and developmental expression profiles of EVI and MDS/EVI during intestinal metamorphosis provide some clues on their likely involvement in adult vertebrate organ development. The T3-dependent amphibian metamorphosis resembles mammalian post-embryonic development, a perinatal period when plasma T3 concentrations are high [1]. During metamorphosis, different organs/tissues are transformed as a tadpole is changed into a frog through largely organ-autonomous processes induced by T3 [2], [3]. Of particular interest is the transformation of the intestine. The tadpole intestine in both *X. laevis* and *X. tropicalis* consists of mostly a monolayer of larval epithelial cells with little connective tissue or muscles [6], [37]. During metamorphosis, the larval epithelial cells undergo apoptosis and concurrently, adult epithelial stem/progenitor cells are formed de novo via dedifferentiation of some larval epithelial cells through yet still-unknown-mechanisms to establish the self-renewal system of the adult intestine [5], [6], [37]–[40]. The connective tissue and muscles also develop extensively, leading to thick layers of these tissues surrounding the adult epithelium at the end of metamorphosis. EVI and MDS/EVI are expressed constitutively in the non-epithelium, which is made of predominantly the connective tissue and muscles, suggesting that EVI and MDS/EVI have no roles specifically for the transformations of these tissues.

The lack or very low level of EVI and MDS/EVI expression in the intestinal epithelium either before or at the end of metamorphosis suggests that they are not required for differentiated larval or adult epithelium. Interestingly, their expression was upregulated by stage 60, the metamorphic climax when plasma T3 is near the maximal level during development [26]. At this stage, morphologically distinct adult progenitor/stem cells begin to be detectable and express genes associated with adult stem cells, such as leucine-rich repeat-containing G protein-coupled receptor 5 (LGR5) [41] and sonic hedgehog [42]–[44]. The expression of EVI and MDS/EVI reaches peak levels at the climax of metamorphosis (stage 61/62) when most of the epithelial cells are adult progenitor/stem cells as the larval epithelial cells have undergone apoptosis. Thus, in the epithelium, EVI and MDS/EVI are most likely expressed specifically in the developing and proliferating adult progenitor/stem cells, arguing for a role in the formation and/or proliferation of these cells.

In addition to its role in adult intestinal development, EVI also appears to be involved in embryogenesis. It is upregulated during *X. laevis* organogenesis, including the developing foregut [45]. Although the expression of MDS/EVI has yet to be analyzed during embryogenesis, it is likely to be upregulated during foregut development based on the similar expression patterns between EVI and MDS/EVI during metamorphosis and their overlapping and sequential expression during formation of the kidney [21]. This is perhaps not surprising since both embryonic organ development and the formation of the adult ones will undoubtedly use many of the same genes.

How EVI and MDS/EVI participate in normal organ-specific adult stem cell development remains largely unknown, outside of hematopoiesis. From studies using hematopoietic stem cells and epithelial cancer cell lines it is clear that EVI can function through multiple epigenetic mechanisms or bind directly to DNA and act as a transcription factor to increase or decrease gene expression [13], [46]. The multiple functional domains found in EVI are conserved and the first zinc finger domain and the CtBP binding domain have been shown to play a critical role in the early formation of *X. laevis* kidney [21]. Though the acidic domain has lower sequence identity among vertebrates, the high number of glutamate and aspartate residues within the region (38% human, 45% *X. laevis*, and 44% *X. tropicalis*, unpublished) is conserved, suggesting the amino acid sequence is less important than the high frequency of acidic residues in this domain. Though the importance of the additional PR domain within MDS/EVI is less clear than the other functional domains and its mechanism of action is still unknown, this domain of MDS/EVI appears to be necessary to maintain quiescent state of long-term hematopoietic stem cells in established adult bone marrow [18]. Its high conservation within vertebrates, including the splice site, further suggests that the PR domain within MDS/EVI has an important function. As both MDS/EVI and EVI are regulated in a similar manner during stem cell formation, it is unclear if either one plays a dominant role, if their function is redundant or supportive, or if they both play unique and important roles.

The induction of the expression of EVI and MDS/EVI by T3 occurs after 1 day of T3-treatment of premetamorphic tadpoles and gradually increases until peaking after 7 days, suggesting that both EVI and MDS promoters could be regulated by T3 via T3 receptor (TR) at the transcription level. Such a direct response would also be consistent with a role of both EVI and MDS/EVI functioning as transcription factors to facilitate the formation of adult progenitor/stem cells during intestinal metamorphosis. Clearly, this raises a number of interesting questions for future studies. As T3 controls metamorphosis by regulating gene expression through TRs [47], [48], it would be important to confirm if T3 activates EVI and MDS genes via TRs directly at the transcription level. If so, does TR bind to a common or separate T3 response elements to regulate the EVI and MDS promoters? Do EVI and MDS/EVI affect intestinal stem cell development by functioning as transcription factors and what are their downstream target genes? Given the similarity and conservations in adult intestinal stem cell development in vertebrates, addressing such questions should provide mechanistic insights on how adult intestinal stem cells are formed during vertebrate postembryonic development.
